# Preparation and Elastic Moduli of Germanate Glass Containing Lead and Bismuth

**DOI:** 10.3390/ijms13044632

**Published:** 2012-04-11

**Authors:** Hj A. A. Sidek, Hamid R. Bahari, Mohamed K. Halimah, Wan M. M. Yunus

**Affiliations:** Department of Physics, Faculty of Science, Universiti Putra Malaysia, 43400 UPM Serdang, Selangor, Malaysia; E-Mails: h_bahari2002@yahoo.com (H.R.B.); halimah@science.upm.edu.my (M.K.H.); mahmood@science.upm.edu.my (W.M.M.Y.)

**Keywords:** glasses, bismuth, lead, germanate, elastic moduli

## Abstract

This paper reports the rapid melt quenching technique preparation for the new family of bismuth-lead germanate glass (BPG) systems in the form of (GeO_2_)_60_–(PbO)_40−_*_x_*–(½Bi_2_O_3_)*_x_* where *x* = 0 to 40 mol%. Their densities with respect of Bi_2_O_3_ concentration were determined using Archimedes’ method with acetone as a floatation medium. The current experimental data are compared with those of bismuth lead borate (B_2_O_3_)_20_–(PbO)_80−_*_x_*–(Bi_2_O_3_)*_x_*. The elastic properties of BPG were studied using the ultrasonic pulse-echo technique where both longitudinal and transverse sound wave velocities have been measured in each glass samples at a frequency of 15 MHz and at room temperature. Experimental data shows that all the physical parameters of BPG including density and molar volume, both longitudinal and transverse velocities increase linearly with increasing of Bi_2_O_3_ content in the germanate glass network. Their elastic moduli such as longitudinal, shear and Young’s also increase linearly with addition of Bi_2_O_3_ but the bulk modulus did not. The Poisson’s ratio and fractal dimensionality are also found to vary linearly with the Bi_2_O_3_ concentration.

## 1. Introduction

Bismuth germanate-based glasses doped with rare-earth oxides gain much attention from researchers due to their potential applications in non-linear optics devices such as optical communication fibers, solid-state lasers, light converters, waveguide, sensors and scintillation detector in positron camera [1–3]. Some optical properties of bismuth in borate, phosphate and silicate glasses were also extensively studied [4].

The lead germanate glasses are also of growing interest, because their promising application including new lasing materials, upconverting phosphors and optical waveguides. All are due to their high density, refractive index, thermal expansion, mechanical strength, high chemical durability, temperature stability as well as with excellent transmission in the infrared (IR) region up to 4.5 micron [5].

Recent studies by Rabukhin and Belousova [6] found that the coordination numbers of cation present in the composition study range does not significantly affect the structural grouping character of the bismuth–containing gallate glasses. However, they noticed that the elastic constants of bismuth-containing borate and germinate are strongly dependent on the change of coordination number of B^3+^ and Ge^4+^.

The photoelastic constants of germanate glasses containing lead and bismuth oxides were studied by Rabukhin [7,8] where he suggested that the photoelastic and elastooptic data could be used for the light and acoustic lines of acoustooptical devices. He proposed that the high photoelastic constants of lead bismuth germanate glass can be achieved through high refractive index and low modulus of elasticity.

For lead borate glass, at low PbO content the formation of four-coordinated boron proceeds at the rate of two tetrahedral for each added oxygen. The lead enters the glass as modifiers Pb^2+^ ions. However, at 15–20 mol% PbO, lead enters the network in the form of PbO_4_ pyramids (with Pb at the apex of the pyramid). These PbO_4_ units bridge prefentially to BO_3_ rather than BO_4_ units [9].

Hamezan *et al*. [10] studied the elastic constants and thermal properties of lead bismuth borate glasses. They found the density and molar volume of such glasses increase with glass modifier content which attributed to the replacement of Bi_2_O_3_ and PbO; both have larger density and molar volume than B_2_O_3_ in the glass networks.

So far, the sound velocity in lead and bismuth containing germanate glass and their elastic constants have not been widely studied. In this research the glassy network of GeO_2_ will be added with PbO and Bi_2_O_3_ glass modifiers. Their elastic properties will be investigated by the ultrasonic pulse-echo technique [11–14]. The present work reports the preparation and ultrasonic characterization of bismuth-lead germanate (BPG) glass systems.

## 2. Results and Discussion

Glass composition of the ternary bismuth lead germanate glasses (BPG) of the ternary form (GeO_2_)_60_–(PbO)_40−_*_x_*–(1/2Bi_2_O_3_) together with density, molar weight and molar volume are given in Table 1. The experimental data of bismuth lead borate (BPB) (B_2_O_3_)_20_–(PbO)_80−_*_x_*–(Bi_2_O_3_)*_x_* [14] is presented for comparison purposes.

**Figure 1:**
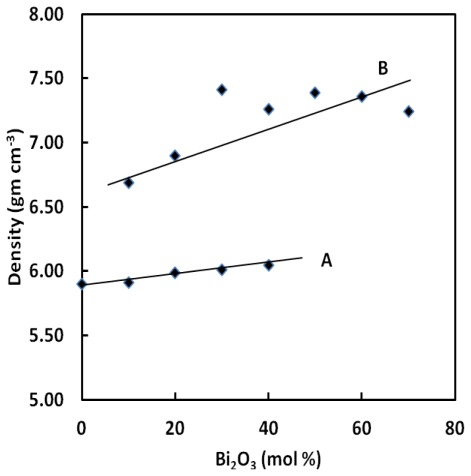
Density of the ternary lead-bismuth germinate glasses of the form (GeO_2_)_60_–(PbO)_40−_*_x_*–(1/2Bi_2_O_3_)*_x_* (A) and compared with those of (B_2_O_3_)_20_–(PbO)_80-_*_x_*–(Bi_2_O_3_)*_x_* (B) [14]. The line is drawn to guide the eye.

It can be seen from Figure 1 that, in contrast to BPB, the density and molar volume of BPG glasses vary monotonically and slowly with the increase of Bi_2_O_3_ content. This change in density by the addition of Bi_2_O_3_ is much related to the change in the atomic mass and atomic of volume constituent elements as given in Table 1. The atomic mass of the Bi, Pb, Ge and B atoms is 208.98, 207.20, 72.63 and 10.811 and their atomic radii are 1.56, 1.75, 1.22 and 0.90 Å respectively. This explains the increase in density with increasing Bi_2_O_3_ content.

Figure 1 shows that densities of all the glass examined are increased with gradual substitution of PbO with Bi_2_O_3_. The ternary borate containing lead and bismuth (B) show higher density changes as compared with those of BPG glasses.

**Figure 2:**
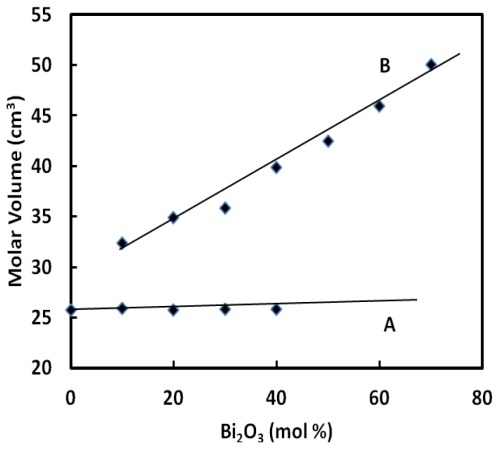
Molar volume of the ternary lead-bismuth germinate glasses of the form (GeO_2_)_60_–(PbO)_40−_*_x_*–(1/2Bi_2_O_3_)*_x_* (A) and compared with those of (B_2_O_3_)_20_–(PbO)_80−_*_x_*–(Bi_2_O_3_)*_x_* (B). The line is drawn to guide the eye.

Both density and molar volume of the glasses increase with an increasing of Bi_2_O_3_. The density and molar volume increases by replacing PbO by Bi_2_O_3_ in Bi_2_O_3_–PbO–GeO_2_ glass system. It can be seen from Figure 1 and Figure 2 that the density of BPG glasses varies from 5.90 to 6.05 g cm^−3^ and the molar volume varies from 25.77 to 25.80 cm^3^ mol^−1^. Generally, the density and the molar volume show opposite behavior but, in this study, this was not the case. In this glass, substitution of lead by bismuth causes the expansion of network. Similar trends for densities and molar volumes have already been reported elsewhere for other glass systems [15–18].

The molar volume of the ternary BPG glasses of the form (GeO_2_)_60_–(PbO)_40−_*_x_*–(1/2Bi_2_O_3_)*_x_* (A) is shown in Figure 2 and compared with those of (B_2_O_3_)_20_–(PbO)_80−_*_x_*–(Bi_2_O_3_)*_x_* (B). It is clear that by increasing Bi_2_O_3,_ the molar volume increases which is similar with the variation density with increasing Bi_2_O_3_ content. For the ternary BPG glasses (A), the change is quite small as compared with the ternary BPB glasses and this might due to the compactness of the glass structure.

The Bi ions may enter the glass network interstitially, hence, some network bonds Ge–O–Ge or Pb–O–Ge are broken and replaced by ionic bonds between Bi ion and singly bonded oxygen atoms. So if one assumed that only effect of adding Bi cations was to break down the network bonds Ge–O–Ge and Pb–O–Ge then an increase in the molar volume with Bi_2_O_3_ content would be expected for the entire vitreous range of the studied glass system. Experimentally, this effect increases the molar volume in the glass compositional range from 0 to 40 mol% Bi_2_O_3_ content (see Table 1) and as a consequence the values of the density are increased.

The addition of Bi_2_O_3_ increased the values of density, which is probably attributable to simultaneous filling up of the vacancies amidst the network by the interstitial Bi ions with atomic mass 208.98. This increase in density indicates a structural change in the glass network which is accompanied by an increase in the molar volume [18,19].

**Figure 3:**
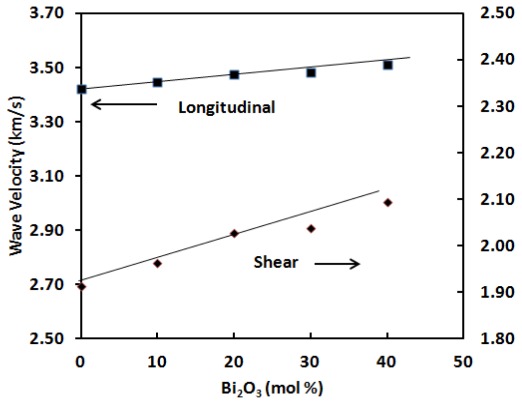
Longitudinal and shear ultrasonic velocities at room temperature in ternary (GeO_2_)_60_–(PbO)_40−_*_x_*–(1/2Bi_2_O_3_)*_x_* glass systems. The line is drawn to guide the eye.

**Figure 4:**
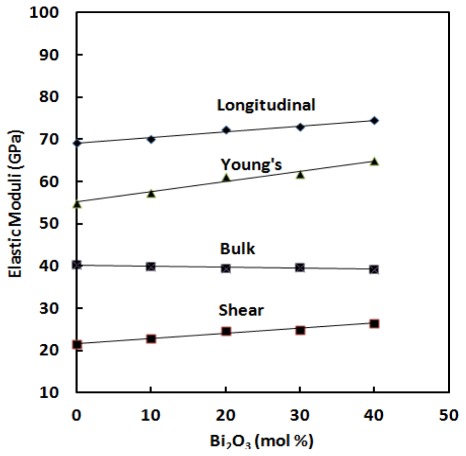
Variation of elastic moduli of ternary (GeO_2_)_60_–(PbO)_40−_*_x_*–(1/2Bi_2_O_3_)*_x_* glass systems with composition. The line is drawn to guide the eye.

The elastic moduli are proportional to the square of velocity and a plot of sound velocities *vs*. composition is indicative of relative structure. The compositional dependence of longitudinal (*V*_l_) and transverse (*V*_s_) sound velocities are depicted in Table 2 and Figure 3. Figure 4 shows the variation of elastic moduli as a function of Bi_2_O_3_ content. All the elastic moduli show the same trend as the ultrasonic wave velocities.

As can be seen from Figures 3,4, both *V*_l_ and *V*_s_, and elastic moduli increase with the addition of Bi_2_O_3_ content over the entire composition studied. The results in Table 2 indicate that the elastic moduli increase linearly with Bi_2_O_3_ content. Longitudinal modulus changes from 69.10 GPa to 74.53 GPa, shear modulus from 21.57 GPa to 26.51 GPa, Young’s modulus from 54.93 GPa to 64.90 GPa and bulk modulus decrease from 40.34 GPa to 39.18 GPa as depicted in Figure 4. The increase in elastic moduli is due to an increase in the cross-link density [20] and therefore an increase in the rigidity of glass samples.

This variation of ultrasonic wave velocities and elastic moduli can be explained on the basis of the structural consideration of germanate glassy network. In the present glass system, their longitudinal ultrasonic velocity increases from 3422 to 3511 ms^−1^ and shear velocity increases from 1912 to 2094 ms^−1^ by increasing of bismuth content. According to Higazy and Bridge [21] the longitudinal strain changes directly with bond stretching force constant. On the other hand, as reported by Reisfeld *et al*. [4] the shear strain changes with bond bending force constant. The increase in velocities is attributed to the increase in rigidity of the glass network.

Glass is considered as elastic substance and, thus, can be characterized through a modulus of elasticity [11,12]. This modulus increases as the lengthening at a certain applied stress diminishes. That will be the case if the glass structure is rigid and therefore contains the fewest possible non bridging oxygen. When an oxide is introduced to germanate, the strength of the structure depends on the field strength of the cation. With increasing Bi_2_O_3_ content in the germanate glass, the structure becomes more rigid and so the density also increase and hence the modulus of elasticity increases [17–19]. It may also be noted from Figure 4 that the rate of change of elastic moduli is more pronounced in longitudinal modulus (*L*) and least in case of transverse or shear modlulus (*G*). This indicates resistance to deformation and it is most probably due to presence of large number of covalent bonds.

Bulk modulus (*K*) is the elastic property of material that can be derived most easily from the glass structure [20]. GeO_2_ posses an open structure characterized by many open spaces. The addition of Bi_2_O_3_ will occupy such spaces and this should leads to an increase in bulk modulus. With low Bi_2_O_3_ contents, the bulk modulus is small since many open spaces are present which will be filled most quickly by the large Bi^3+^ ions (ion radius is 1.56 Å), so that the bulk modulus increases. With higher Bi_2_O_3_ contents, the ability for deformation of the cations becomes influential. This is clear from Figure 4, which shows the relation between bulk modulus and Bi_2_O_3_ content.

**Figure 5:**
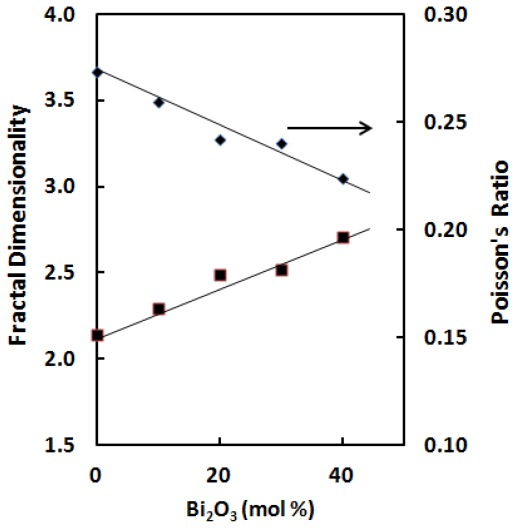
Variation of fractal dimensionality and Poisson’s ratio of ternary (GeO_2_)–(PbO)–(1/2Bi_2_O_3_) glass systems with composition. The line is drawn to guide the eye.

The compositional dependence of Poisson’s ratio and fractal dimensionality parameter as a function of Bi_2_O_3_ content is given in Figure 5. For BPG glasses their Poisson’s ratio, the ratio of transverse and linear strains for a linear stress, decrease from 0.27 to 0.22. Poisson’s ratio has also been discussed in terms of the dimensionality of glass network and it is observed that the Poisson’s ratio for a three dimensional network is less than that of a two dimensional structure, which in turn is less than that of a one dimensional structure. The decrease in Poisson’s ratio implies to the increase of crosslink density of the glass as proposed by Higazy and Bridge [21].

The fractal dimensionality parameter (*d* = 4*G*/*B*) listed in Table 2 suggest that in all glasses the dimensionality of glass structure lies between 2.14 and 2.71. As it can be seen from Figure 5, the Poisson’s ratio is found to nonlinearly decrease with an increase of Bi_2_O_3_ content. Further, the values of Poisson’s ratio are believed to be that of a covalently bonded structure. With an increase of Bi_2_O_3_ concentration, an increase in fractal dimensionality is observable. This suggests that, by increasing bismuth cations, dimensionality of BPG glass samples increase.

## 3. Experimental

The new family of bismuth lead germanate (BPG) glasses in form of (½Bi_2_O_3_)*_x_*–(PbO)_40−_*_x_*–(GeO_2_)_60_ where *x* = 0 to 40 wt% have been successfully prepared by a rapid melt quenching technique [22]. The reagent grade raw materials (99.9% purity of GeO_2_, PbO and Bi_2_O_3_) were weighed out the ratio of the specified composition (in batches of approximately 15 g for each composition) as presented in Table 1.

The raw materials were mixed carefully in an agate mortar and then the mixtures in alumina crucible were preheated in the first furnace at a temperature of 300 °C for 30 min. The mixtures were then transferred in a second furnace and melted in 1100 °C for 1 h under atmospheric conditions. The mixtures were stirred regularly to obtain a better mixing of the composition and to improve the homogeneity of the glass samples.

The melt was then poured quickly on a preheated split metal mold and transferred to the first furnace for annealing at 420 °C before cooling down to room temperature for 24 h. The glass samples with 12 mm diameter and 30 mm thickness were bubble free and homogeneous with good optical quality. A similar procedure was employed to prepare other glass samples. The color of glass sample varies from light to dark violet as more Bi_2_O_3_ were added into germanate glassy networks.

The density (*ρ*) of the glasses were determined by Archimedes method with acetone as buoyant liquid using the relation

$$\rho =\left(\frac{{\text{W}}_{\text{a}}}{{\text{W}}_{\text{a}}-{\text{W}}_{\text{b}}}\right)\rho_{b}$$

where *W*_a_ is the glass sample weight in air, *W*_b_ the glass sample weight in buoyant and *ρ*_b_ the density of the buoyant. All the glass sample’s weights were measured with a digital balance (±0.0001 g accuracy). Their molar volume was calculated from the molecular weight (*M*) and density (*ρ*). The accuracy in the measurement of the density is ±0.01 g cm_−3_ and the relative error is ±0.05%.

The chemically estimated elemental composition values present in the glass samples with the Atomic Absorption Studies (Perkin–Elmer, Model 1372, USA) were found to be slightly smaller than the corresponding elemental nominal composition values (before glass formation), which we considered to be due to evaporation losses and uncertainties in the chemical analysis.

All the glass samples were checked by X-ray diffraction for their amorphous nature using X’Pert Pro Panalytical PW 3040 MPD X-ray powder diffractometer by employing Cr-K*a* radiation. The absence of any crystalline peaks in the XRD patterns of the present glass samples indicates the amorphous nature.

For the measurements of ultrasonic velocity in bismuth lead germanate (BPG) glass samples, the samples were shaped into a circular disc of 12 mm diameter and 10–12 mm thickness. The opposite faces of the disc shaped glass samples were highly polished using very fine lapping papers to achieve a good surface finish with plane parallelism having accuracy of ±5 micron.

Ultrasonic velocity measurements were carried out at a frequency of 10 MHz using x-cut and y-cut quartz transducers. A pulse superposition technique was employed using Ultrasonic Data Acquisition System (MATEC 8020, Matec Instruments, USA) [12,22]. Burnt honey was used as a bonding material between the glass samples and transducers. By measuring the thickness of the sample (*d*), longitudinal (*V*_l_) and transverse (*V*_t_) wave velocities were calculated using the relation, *V* = 2*d*/*t* [12,13]. The absolute accuracy in the measurement of the velocity is ±5 ms^−1^ and the relative error is ±0.1%.

Glasses are isotropic and have only two independent elastic constant of *L* and *G* which are obtained from longitudinal and shear sound wave velocity and density of the present glass samples. The various elastic properties of the glasses were calculated using the following relations [22]:

(1) $$\text{Longitudinal modulus}:\;L=\rho {V_{1}}^{2}$$

(2) $$\text{Shear modulus}:\;G=\rho {V_{\text{s}}}^{2}$$

(3) $$\text{Bulk modulus}:\;K=\rho \left({V_{l}}^{2}-\frac{4}{3}{V_{s}}^{2}\right)$$

(4) $$\text{Young}’\text{s modulus}:\;E=\frac{\rho {V_{s}}^{2}(3{V_{l}}^{2}-4{V_{s}}^{2})}{{V_{l}}^{2}-{V_{s}}^{2}}$$

(5) $$\text{Poisson}’\text{s ratio}:\;\sigma =\frac{\left({V_{l}}^{2}-2{V_{s}}^{2}\right)}{2({V_{l}}^{2}-{V_{s}}^{2})}$$

(6) Fractal dimensionality: d=4G/K

## 4. Conclusions

New ternary germanate based glass samples added with bismuth and lead oxides were successfully prepared and their glassy natures were confirmed by the XRD method. Based on the result obtained, it demonstrated that the density and molar volume increase with glass modifier content, which more attributed to the replacement of Bi_2_O_3_ and PbO; both had larger density and molar volume than germanate glass networks. Such increase in their density and volume with composition are due to the compactness of the structure. The observed higher values in velocity at high modifiers content for this glass types, confirmed a substantial change in glass structure. Their longitudinal, shear and Young’s modulus for bismuth lead germanate glasses are also found to increase with the addition of Bi_2_O_3_. Meanwhile, there was a similar pattern in elastic moduli with the increasing of Bi_2_O_3_ content, where the values of both ultrasonic wave velocities increased subsequently.

## Figures and Tables

**Table 1:** Glass composition of the ternary lead-bismuth germanate glasses of the form (GeO_2_)_60_–(PbO)_40−_*_x_*–(1/2Bi_2_O_3_)*_x_* together with density, molar weight and molar volume. The experimental data of (B_2_O_3_)_20_–(PbO)_80−_*_x_*–(Bi_2_O_3_)*_x_* [14] is included for comparison.

Samples	Glass Composition (mol%)	Density (g cm^−3^)	Molar Weight (g/mol)	Molar Volume (cm^3^)
	GeO_2_–PbO–1/2Bi_2_O_3_			
A1	60–40–0	5.90	152.06	25.77
A2	60–30–10	5.91	153.04	25.89
A3	60–20–20	5.99	154.01	25.73
A4	60–10–30	6.01	154.99	25.78
A5	60–0–40	6.05	155.97	25.80
	B_2_O_3_–PbO–Bi_2_O_3_			
B1	20–70–10	6.69	216.76	32.40
B2	20–60–20	6.90	241.04	34.93
B3	20–50–30	7.41	265.31	35.80
B4	20–40–40	7.26	289.59	39.89
B5	20–30–50	7.39	313.86	42.47
B6	20–20–60	7.36	338.14	45.94
B7	20–10–70	7.24	362.42	50.06

**Table 2:** The room temperature ultrasonic wave velocities and elastic moduli of the ternary lead-bismuth germanate glasses together with fractal dimensionality and Poisson’s ratio.

Glass sample	*x*	Velocity (m/s)		Elastic Moduli (GPa)				*d* = 4*G*/*K*	Poisson’s Ratio

		*V*_l_	*V*_s_	*L*	*E*	*K*	*G*		
A1	0	3422	1912	69.10	54.93	40.34	21.57	2.14	0.27
A2	10	3445	1963	70.16	57.39	39.79	22.78	2.29	0.26
A3	20	3475	2027	72.28	61.10	39.49	24.59	2.49	0.24
A4	30	3482	2037	72.89	61.86	39.63	24.95	2.52	0.24
A5	40	3511	2094	74.53	64.90	39.18	26.51	2.71	0.22
